# Mechanosensing, from forces to structures

**DOI:** 10.3389/fpls.2022.1060018

**Published:** 2022-12-01

**Authors:** Feng Zhao, Yuchen Long

**Affiliations:** ^1^ Collaborative Innovation Center of Northwestern Polytechnical University, Shanghai, China; ^2^ School of Ecology and Environment, Northwestern Polytechnical University, Xi’an, Shaanxi, China; ^3^ Department of Biological Sciences, The National University of Singapore, Singapore, Singapore; ^4^ Mechanobiology Institute, The National University of Singapore, Singapore, Singapore

**Keywords:** morphogenesis, mechano-sensation, mechano-heterogeneity, mechano-transduction, arabidopsis

## Abstract

Sessile plants evolve diverse structures in response to complex environmental cues. These factors, in essence, involve mechanical stimuli, which must be sensed and coordinated properly by the plants to ensure effective growth and development. While we have accumulated substantial knowledge on plant mechanobiology, how plants translate mechanical information into three-dimensional structures is still an open question. In this review, we summarize our current understanding of plant mechanosensing at different levels, particularly using Arabidopsis as a model plant system. We also attempt to abstract the mechanosensing process and link the gaps from mechanical cues to the generation of complex plant structures. Here we review the recent advancements on mechanical response and transduction in plant morphogenesis, and we also raise several questions that interest us in different sections.

## Introduction

Plants, like other living organisms, are constantly exposed to various stimuli from their environments, including mechanical stimuli. Being relatively sessile compared to animals, plants have evolved different strategies in response to these stimuli. The mechanical stimuli that affect plants could be classified into two types: contact (e.g. rain, wind, touch by another organism, etc.), and non-contact (e.g. gravity, electromagnetic force, etc.) forces. Whatever the source of stimuli, plants do not only passively adapt to the outside ‘risks’, but rather actively respond to these stimuli by incorporating their intrinsic machinery to reproducibly shape themselves, and in turn sculpt the ecosphere.

Morphogenesis is a multiscale process, involving complex feedback between genetic, biochemical and biomechanical regulations. Plant cells cannot move as they are constrained by rigid extracellular matrices or “cell walls”. Thus, plant morphogenesis mainly depends on growth regulations, specifically in growth rates and directions. The force that drives growth is ultimately generated by turgor pressure, which is borne and harnessed by the cell wall. The plant primary cell wall is a complex amalgamation of various polysaccharides, glycoproteins, and water, and is known for its remarkable properties to both withstand mechanical forces and being pliable to grow. The design principle behind such property has recently been intensively reviewed by Cosgrove (2022).

During growth, the walls yield to the mechanical stresses caused by turgor pressure, allowing the cells to change their shape. Cell wall extensibility and stiffness are found to be associated with growth rates, and growth direction is often found regulated by wall anisotropy. Good examples are the largely anisotropic cell wall stiffness patterns in the elongating *Arabidopsis* hypocotyl (Peaucelle et al., 2015) versus the largely isotropic cell walls of the organ primordia in the *Arabidopsis* shoot apical meristem (SAM) (Sassi et al., 2014). Specific cell shapes in turn prescribe specific stress patterns, which could be sensed by cells. This “mechanical feedback” between force and shape has been well-known to channel several morphological events (Hamant et al., 2008; Hervieux et al., 2016; Zhao et al., 2020; Trinh et al., 2021). Additionally, thanks to the heterogeneous property of cell wall mechanics, the stress intensity and direction appear very noisy at the tissue or even larger scale. Given the plant cells are connected by the cell-cell adhesion and plasmodesmata, the local mechano-heterogeneity could be sometimes buffered or oppositely amplified (Uyttewaal et al., 2012; Hong et al., 2018). How do plants coordinate the fluctuations coming from external and internal mechanical stimuli and reproduce robust structures? To discuss this complex issue, we will first look at how plants sense external cues.

## Mechanical sensation– from external to internal signals

### Touch

Touch is a contact force. In theory, wind, rain, hail, tides, and touch by other organisms should all trigger touch responses. Hence, the reaction to touching is very general and complex in plants. In some plants, the touch response could be very fast (within 1 second), and it is usually associated with specialized structures. For example, the trigger hairs on the Venus Flytrap’s leaves and the motor cells of *Mimosa pudica* are well-known structures for sensing and conducting fast responses. Similarly, the Arabidopsis leaf trichomes are also reported as fast touch signal transduction sensors (Matsumura et al., 2022).

How do plants sense touch? Often, the site of force sensing can be less defined, as demonstrated by experiments of brushing (Jensen et al., 2017), wounding (Hamant et al., 2008), stretching and compressing (Robinson and Kuhlemeier, 2018). Other touch responses may involve specialized structures mentioned above. For example, hair-like structures have high aspect ratios (length: diameter) and are easy to bend, the causal longitudinal strain perception is known critical for touch sensitivity (Seale et al., 2018). At the molecular level, several mechanisms have been proposed. One hypothesis suggests squeeze-generated cytosolic stream is essential for the perception of touch, evidenced by the moving of organelles upon stimulations (Chehab et al., 2009). A second hypothesis suggests stretch-activated channels are responsible for triggering touch sensing, where touch-induced bending may trigger asymmetric stretching on the outside and the inside of the bend. This idea is further supported by a recent study showing the expression of mechanosensitive ion channels in the Venus flytrap trigger hairs (Procko et al., 2021). The third possibility suggests that touch results in the perturbation of the cell wall-plasma membrane-cytoskeleton continuum (Chehab et al., 2009).

Touch simultaneously induces a deformation of the plant, or strain, and a stress pattern associated to it. At the cellular level, how strain and stress are sensed upon touching is still poorly understood (Fruleux et al., 2019). Periodic touch stimuli could lead to altered plant growth, like reduced elongation, excess lateral expansion of stems, and delayed flowering. This phenomenon was first coined by Mark Jaffe (Jaffe, 1973) as thigmomorphogenesis, which is a relatively slow process over days to weeks. It has been known for a long time that, many touch-inducible genes were associated with intrinsic mechanical responses, such as Ca^2+^ signaling (like calmodulin-like, CML genes), hormones (like ethylene), and cell wall metabolism (like xyloglucan endotransglucosylase/hydrolase, XTH genes) (Braam, 2005). Recent research nicely presents a link from external touching to internal signaling pathway, showing plants incorporate ethylene-dependent pectin degradations to tune their own shape (Wu et al., 2020).

### Gravity

Compared to touch, gravity is a non-contact force. How plants react to gravity is a long-standing biological question. When we talk about gravity response, we usually discuss growth directionality relative to the gravity vector, alias gravitropism. Gravitropism is a complex process, which could be simplified into three stages: perception, signaling transduction, and bending. Gravity is believed to be mainly sensed within specific cell types: columella in the root, and endodermis in the shoot (Kawamoto and Morita, 2022). In the classical starch-statolith hypothesis (Sack, 1997), the direction of gravity is proposed to be sensed by the sedimentation of statoliths (starch-filled amyloplasts) in columella and endodermal cells, where the weight of statolith is either sensed by the cell edge or cytoskeleton (e.g. Perbal and Driss-Ecole, 2003; Leitz et al., 2009). Recent studies have revealed the complexity of statolith movement, showing that the statoliths behave more like an active liquid than a granular material (Bérut et al., 2018), and that shoot bending is insensitive to a range of gravity intensities artificially applied by centrifugation but to the inclination angles between the shoot and the gravitational vector (Chauvet et al., 2016). Contrarily, another series of hypotheses (including the gravitational pressure hypothesis (Staves, 1997); tensegrity hypothesis (Ingber, 1997), etc.) suggested that gravity is sensed generally by all cell types, and the cell wall-plasma membrane-cytoskeleton continuum can act as a force-transmission scaffold to transduce mechanical signals into biochemical response.

Upon gravity sensing, several biochemical signal transductions are activated. Among these signals auxin is prominent in modulating differential growth and organ bending (Takahashi et al., 2021). Gravitropic bending is actually a biphasic process: the first phase is curving towards gravity axis, the second phase is a straightening process called autotropism (Bastien et al., 2013). Intriguingly, the second decurving process is gravity-independent. Recent research shows that actin cables in stem fiber cells perform as plant posture sensors. This intrinsic geometric sensing is essential for gravitropism (Okamoto et al., 2015; Moulia et al., 2021).

## Mechano-heterogeneity

In multicellular plants, cells are glued together to form a continuity (Atakhani et al., 2022). However, the mechanical response for each cell is not necessarily homogeneous. Heterogeneity is an intrinsic property of the biological system, spanning all aspects from molecular to organism levels. Therefore, how a robust order is built up during plant morphogenesis is a fascinating question.

### Cell wall heterogeneity

Being a fiber-enforced complex structure, the plant cell wall is far from homogenous. Several key compounds and structures in the cell wall have been proposed to mediate its physical properties during morphogenesis. For example, the stiff cellulose microfibrils can be randomly arranged or aligned into directional arrays. Microfibril anisotropy is thought to be laid down primarily during synthesis by the cellulose synthase complex (CSC) tracing along existing microfibrils or the underlying cortical microtubule (CMT), which were shown to align to the direction of the principal tensile stress (Hamant et al., 2008; Chan and Coen, 2020). Anisotropic microfibril arrays can then fortify cell walls in the same direction to restrict growth, biasing growth anisotropy in the perpendicular direction (Jonsson et al., 2022). Pectin forms matrices in the cell wall, and its methylesterification state and binding to Ca^2+^ can modulate the matrix stiffness to modulate cellular growth (Du et al., 2022). Recently, it was proposed that pectin can form nanofilaments, which are local heterogeneities of methylated/demethylated pectin foci, and their unequal swelling may contribute to the undulated shape of Arabidopsis pavement cells (Haas et al., 2020).

Cell wall synthesis and modifications are highly local during development (Dauphin et al., 2022), which implies that cell wall mechanical properties can vary spatiotemporally. Indeed, direct mechanical measurements revealed that cell wall moduli, a measure of material stiffness, is highly patchy from subcellular to tissue levels (reviewed by Hong et al. (2018)). One may expect such “noisy” cell wall patterns should introduce growth heterogeneity and reduce organ shape reproducibility. Surprisingly, however, plants may alternate the stiff and soft patches over time by “spatiotemporal averaging”, as proposed in the Arabidopsis sepal (Hong et al., 2016). Long-range coordination between patches dampens local variability, but counterintuitively reduces the effect of the “spatiotemporal averaging, thereby reducing organ shape reproducibility (Hong et al., 2016). This example demonstrates how local mechanical heterogeneity may contribute to global shape robustness.

The biochemical reactions between wall polymers are proposed to account for cell wall’s macroscopic mechanical properties (Zhang et al., 2021; Dumais, 2021). These reactions also occur over time, and together with the laminar deposition of new cell wall materials, may create gradients of stress and strain on top of the patchiness along the thickness of the wall (Lipowczan et al., 2018). One may picture this as a “fractal of heterogeneity”: existing patchy cell wall expands differentially and gets larger, and a new layer of cell wall with smaller patches is added underneath the previous layer, and repeats. Over time, this temporal accumulation can lead to heterogeneity on many lengthscales, which may contribute to the different levels of local growth variability and long-range coordination in plant tissues (Fruleux and Boudaoud, 2021).

### Heterogeneity arising from cell-cell interaction

Arabidopsis pavement cells acquire interlocked jigsaw shapes, generated by coordinated, heterogeneous growths within and between the cells (Elsner et al., 2018). Besides the well-established molecular pathways involving auxin and Rho GTPases, biomechanical heterogeneity has been considered as another candidate for shape generation [reviewed by Liu et al. (2021)]. The aforementioned mechanical feedback theory can explain shape acquisition for single pavement cells (Sampathkumar et al., 2014; Sapala et al., 2018), particularly in reinforcing and amplifying initial minor undulations of the cell. However, pavement cells are not isolated, and one cell’s lobe must match its neighbor’s neck. It was found that anticlinal cell walls between two neighboring pavement cells can have asymmetric pectin deposition and different moduli, which precedes lobe-neck formation and would lead to bending either under tension or when pectin expands upon demethylation (Majda et al., 2017; Haas et al., 2020). Another study proposes that mechanical asymmetry in anticlinal walls is not sufficient for pavement cell shape, and stress pattern in the outer periclinal wall is further required to generate the matching puzzle shapes of the pavement cells: by including periclinal walls in mechanical simulations, turgor pressure may cause the cell edge to buckle. The resulting stress heterogeneity may initiate local cytoskeleton reorganization, which will promote lobe-neck formation (Bidhendi and Geitmann, 2019). The attractive part of this model is that, the regular wavelength of the buckling may emerge from cell geometry and mechanical properties like bending stiffness, potentially giving rise to predictable heterogeneity that contributes to complex cell shape formation without initial prepatterning. All these, coupled with local growth heterogeneity and the microtubule-CSC-mediated mechanical feedback, likely underlie the generation of the convoluted pavement cell geometry (Belteton et al., 2021). In a more complex three-dimensional context, a recent model suggested that different mechanical feedback activity in inner and outer cell walls of laminar organ primordia ensures the cortical microtuble-mediated growth anisotropy for organ flattening (Zhao et al., 2020).

Other kinds of mechanical heterogeneity can also emerge from tissue arrangements. For example, cell arrangements can contribute to heterogeneity in apparent stiffness on both cell and tissue levels (Mosca et al., 2017; Majda et al., 2022). Turgor pressure, which was often believed to be homogenous between plant cells connected by plasmodesmata, was recently demonstrated to be heterogeneous in the epidermis of Arabidopsis shoot apical meristem (SAM). Unlike the heterogeneity of cell wall moduli, which appears random, turgor pressure level anticorrelates with cell size and cell neighbor number in the epidermis (Long et al., 2020). This pressure pattern is reminiscent of the pressure distribution in soap bubbles (Weaire and Hutzler, 2001), which emerges from cell geometry and topology. This, and the buckling theory above, hint that biomechanical variability may not always be random; the pattern may be predictable, and they may serve as the symmetry-breaking event for subsequent growth patterning. For further discussion on variability versus stochasticity, see Long and Boudaoud (2019).

## Mechanical signaling transduction

Considering all challenges posed above, how is stress or strain being sensed by plants, and what’s the molecular mechanism behind? In the last decades, we accumulated substantial knowledge about mechanical signaling transductions. Exploring the underlying mechanism may help us to get a better understanding of how plants react to forces.

### Mechano-sensors

A plant cell could be conceptually described as a plasma membrane (PM) wrapped water balloon constrained by the rigid cell wall. As mentioned above, cell wall is the main load-bearing component in a plant cell, and the trigger for cell wall mechanical responses could be stress, strain, or both (Fruleux et al., 2019). At the molecular level, a large protein family–receptor-like kinase (RLK) is generally thought to be important for cell wall mechano-sensing. The most well-studied RLKs in mechano-transduction are FERONIA (FER) and THESEUS1(THE1). FER is multifunctional. For instance, FER is a negative regulator in touch response (Darwish et al., 2022), whereas actively regulates the F-actin mediated PIN polar localization in gravity response (Dong et al., 2019). Interestingly, FER could interact with pectin to activate ROP GTPase and thus regulates pavement cell morphogenesis (Tang et al., 2022; Lin et al., 2022), and also contributes to the mechanical sensing in shoot morphogenesis in a microtubule-independent manner (Malivert et al., 2021). By contrast, THE1 is first identified as a wall integrity sensor by pharmacological screen, and recently reported to be responsible for coordinating changes in turgor pressure and cell wall stiffness (Bacete et al., 2022).

In normal conditions, the rigid cell wall resists the internal turgor pressure and reaches an equilibrium. In definition, turgor is the force within the cell that pushes the plasma membrane against the cell wall. The turgor thus must be surveilled constantly in order to keep the wall and membrane integrity. Indeed, a class of proteins named mechanosensitive (MS) ion channels is important for membrane tension perception. For example, Ca^2+^ channels like OSCA relies on tension sensing and mediates osmosensing in Arabidopsis (Yuan et al., 2014), and intrinsically disordered region (IDR) proteins like AtLEA4 are important for the response to increased osmolarity (Cuevas-Velazquez et al., 2021). For a detailed review on osmotic sensing, referred to Gorgues et al. (2022).

Whereas the importance of cell wall and PM in mechano-sensing is obvious, more and more evidence supports the rational existence of a physical continuum from cell wall to PM, cytoskeletons, and even internal organelles (Lang et al., 2004; Fal et al., 2017; Hamant et al., 2019; Lee et al., 2019; Yoneda et al., 2020; Goswami et al., 2020a; Goswami et al., 2020b; Fal et al., 2021; Schneider et al., 2021; Codjoe et al., 2022; Radin et al., 2022). Once the force transmission chain is a continuum at the cellular level, where are the hotspots for the connection among different compartments is still an open question.

In theory, the propagation of force is very fast in stiff tissues. Thus, mechanical forces could reasonably act as long-distance signals to cooperate plant growth and morphogenesis at tissue or even organ level. On such a larger scale, cells are connected *via* plasmodesmata and cell-cell adhesions. Interestingly, plasmodesmata and cell adhesions are recently reported as important components for the coordination of tensile stress and tissue patterning at the supracellular level (Verger et al., 2018; Hernández-Hernández et al., 2020). While supracellular mechanical signaling has been shown to be important for plant growth and morphogenesis (Zhao et al., 2018; Long et al., 2020; Moulia et al., 2021; Trinh et al., 2021), how the cellular mechano-sensing is coordinated at a larger scale is still poorly understood.

### Signaling transduction

Force transduction must include the interaction with chemical signals. Recently, some nexuses of force and chemical signaling transduction are emerging. Ca^2+^-mediated signaling, hormones (such as auxin, ethylene, JA, GA), ROS, and immune response are all involved for example in touch response (Li et al., 2019; Wu et al., 2020; Darwish et al., 2022; Matsumura et al., 2022). A clear picture emerges: once the machinal signals arrive, there must be a very fast response (like electric and Ca^2+^ signaling) to the stimuli, (e.g., Nguyen et al., 2018; Hagihara et al., 2022), after that, the components related to energy, material production, and modifications are mobilized to support the whole system. The feedback is thus essential.

## Discussion

In this review, we take touch and gravity perception as two examples, representing contact and non-contact environmental forces respectively, to glimpse how these forces shape plant organs, and how plants in turn feed back to these physical stimuli. In addition, we also discussed how mechanical heterogeneity may arise from stochasticity or emerge from tissue geometrical arrangements. It is likely that tissue mechanical heterogeneity may introduce further complexity and refinement to the local and global perceptions of external mechanical stimuli, which may then trigger different morphological responses, forming yet another feedback that integrates information across various scales. Lastly, and in response to this proposition, we try to find the nexus point of extrinsic and intrinsic mechanical responsive pathways.

There are many outstanding questions. First, how do plants distinguish different mechanical cues? One apparent strategy/solution is to form specific tissues or organs. This raises another connected question: do mechanical cues contribute to the specification of these cells? Recently, several studies indicated how mechanical forces are upstream of cell fates specification (Landrein et al., 2015; Fal et al., 2016; Hernandez-Lagana et al., 2021), although there are still large spaces for further exploration; Second, how do plants embrace the seemingly contradictory heterogeneity and robustness? Regarding this intriguing topic, readers could refer to Hong et al. (2018); Third, where and how are the mechano-chemical hotpots formed? This is relevant to cell and organ polarity, which is also a kind of heterogeneity. For polarity and mechanics, readers could refer to Gorelova et al. (2021); The last, on a large scale, how the plant shapes are evolutionally preserved and finally fixed under environmental force pressure is also not fully understood.

## Author contributions

FZ and YL contributed equally. All authors contributed to the article and approved the submitted version.

## Funding

This work was supported by the Basic Research Field Project “Science and Technology Innovation Action Plan” of the Shanghai Science and Technology Commission (22JC1402800) and the Fundamental Research Founds for the Central University (D5000220167) to FZ, and a Ministry of Education Academic Research Fund Tier 1 grant (MOE AcRF 21-0065) to YL.

## Acknowledgments

We thank Ali Ferjani for the invitation to write this review. We apologized to those authors whose excellent work was not included in this review due to the word limit.

## Conflict of interest

The authors declare that the research was conducted in the absence of any commercial or financial relationships that could be construed as a potential conflict of interest.

## Publisher’s note

All claims expressed in this article are solely those of the authors and do not necessarily represent those of their affiliated organizations, or those of the publisher, the editors and the reviewers. Any product that may be evaluated in this article, or claim that may be made by its manufacturer, is not guaranteed or endorsed by the publisher.
